# Blue, Yellow, and Red Carbon Dots from Aromatic Precursors for Light-Emitting Diodes

**DOI:** 10.3390/molecules28072957

**Published:** 2023-03-26

**Authors:** Zhenzhen Liu, Xiaofei Lu, Menglin Liu, Wenjing Wang

**Affiliations:** 1 College of Chemistry and Chemical Engineering, Shandong Sino-Japanese Center for Collaborative Research of Carbon Nanomaterials, Instrumental Analysis Center of Qingdao University, Qingdao University, Qingdao 266071, China; liuzhenzhenlz@163.com (Z.L.); l13685407207@163.com (X.L.); 2 Rizhao Custom, Rizhao 276826, China; liumenglin_neu@163.com

**Keywords:** carbon dots, multicolor emission, fluorescence mechanism, light-emitting diodes

## Abstract

In this work, multicolor fluorescent carbon dots with red (R-CDs), yellow (Y-CDs), and blue (B-CDs) emissions were prepared by choosing proper aromatic precursors with different amounts of benzene rings through a simple solvothermal method. The characterization showed that the prepared carbon dots were spherical with a size under 10 nm, rich surface functional groups, and good stability. The emission wavelengths were located at 440, 530, and 580 nm under the excitation of 370 nm. The relative fluorescence quantum yield (*QY*) of R-CDs, Y-CDs, and B-CDs was 11%, 59%, and 33%, respectively. The related characterization demonstrated that the redshift in the photoluminescence was caused by the synergistic effect of the increasing graphitic nitrogen content, quantum size effect and surface oxidation state. By mixing the three prepared CDs into a PVA matrix, the transparent and flexible films produced relucent blue, yellow, and red emissions under 365 nm UV light, and solid-state quenching was effectively avoided. LEDs were fabricated by using B-CDs, Y-CDs, and R-CDs/PVA with a semiconductor chip. These CDs-based LEDs produced bright blue, yellow, and red light with CIE color coordinates of (0.16, 0.02), (0.38, 0.58), and (0.50, 0.49) were successfully manufactured utilizing the prepared blue, yellow and red multicolor carbon dots as the solid luminescent materials. The results showed that the synthesized CDs can be potentially applied in multi-color monitors as a promising candidate for light-emitting diodes (LEDs). This work blazes a novel trail for the controllable preparation of multicolor fluorescent carbon dots.

## 1. Introduction

Carbon dots (CDs), as neoteric carbon nanomaterials, have the unique properties of excellent photoluminescence (PL) emission, low toxicity and cost, high biocompatibility, and easy functionalization [1,2], and can be applied in the fields of optical devices [3,4], biomedicine [5], sensing [6,7,8], imaging [9], and catalysis [10].

The tunable and multicolor PL property is so fascinating that researchers are making great efforts to synthesize, modify, and functionalize CDs in order to regulate and control the emission of CDs. The multicolor emission can be adjusted by the excitation of the wavelength, pH, the concentration, solvent used, carbon source, reaction condition, separation, and pressure [11,12]. Reaction conditions play a vital role in the synthesis of MCDs. Reaction conditions include changing the response temperature [13], time [14], reactant ratio [11], reaction solvent type [15,16], and pH [17], or using machine learning assistance [18]. Among them, the reaction temperature influences the carbonization of carbon resources, then affects the degree of graphitization [1,13]. During synthesis, the reaction time influences the degree of the completion of MCDs, then affects the surface oxidation and size of the CDs [14]. The reaction solvent will control the degree of carbonization and dehydration in the carbon precursors, and then affects the size of the sp^2^-conjugated domains [16]. The surface functional groups of CDs can be directly affected by the reactant ratio, affecting the surface energy levels and causing electron transfer [11]. Varying kinds of carbon precursors produce different CD carbon core hybridization and surface functional groups [12]. The dispersion environments of MCDs, e.g., pH and solvents, cause a hydrogen bonding effect or π–π stacking interaction [11,12].

Currently, photoluminescence mechanisms are mainly divided into a quantum size effect, a molecular state effect, a surface state effect, surface defects, and a cross-linked enhanced emission effect [12,19]. Furthermore, the structures, properties, and photoluminescence mechanisms of MCDs are varied depending on their synthesis strategies or carbon precursors.

However, due to the unclear photoluminescence mechanism and complex structure of CDs, the preparation methods are different and there are no rules to follow, which both limit the further application of CDs. The regulation of the emission of CDs is perplexing and needs to be improved.

Multicolor CDs can be applied in the fields of biological imaging [4,9], sensing [20], and anti-counterfeiting [21]. Light-emitting diodes (LEDs), which are widely used in industrial production, manufacturing, and in households, have attracted great attention recently because of their long life, low power dissipation, small size, and high current-to-light efficiency [22,23,24,25]. More recently, multicolor carbon dots have emerged as promising candidates for LED applications due to their low toxicity, stability, and ease of preparation [17,19,26,27,28]. However, it is urgent to prevent solid quenching caused by π–π aggregation. Moreover, long wavelength emission, especially in red emissive carbon dots, is still a key challenge. It is of vital importance to seek appropriate carbon precursors, surface functionalization methods and preparation methods that are simple and systematic, in order to obtain multicolor carbon dots.

CDs are composed of a conjugated graphite nuclei and a surface with functional groups. The conjugation length and sp^2^ domain size of the carbon core, and the surface functional groups of the surface, have a great impact on the redshift of the emission wavelength. Based on this, in this study, a new synthesis method is constructed in order to prepare multicolor emissive CDs via adjusting the effective conjugation length and the number of surface functional groups. The emission of CDs is effectively modulated by choosing different carbon precursors. To be specific, the fluorescence emission wavelength of CDs can be adjusted from 410 to 580 nm by controlling the extent of graphitic N and the amount of surface -COOH functional groups. The relative photoluminescence quantum yields of B-, Y-, and R-CDs reach up to 33%, 59%, and 11%, respectively. We further fabricate a flexible CDs/PVA film, which can be applied as blue, yellow, and red phosphor powders, and encapsulate a ground substance for multicolor LEDs or white LEDs. By assembling the B-CDs/PVA, Y-CDs/PVA, and R-CDs/PVA with semiconductor chips, a blue LED with a Commission on Illumination (CIE) coordinate (0.16, 0.02), a yellow LED with a CIE coordinate (0.38, 0.58) and a red LED with a CIE coordinate (0.50, 0.49) were fabricated successfully.

## 2. Results and Discussion

### 2.1. Preparation and Characterization of the Prepared CDs

According to the photoluminescence mechanism of CDs, the conjugation length and the sp^2^ domain size influence the emission. Therefore, considering the conjugation structure of benzene ring, we choose three typical aromatic precursors (aniline, 1,8-diaminonaphthalene, and [1,1′-binaphthalene]-2,2′-diamine) with different benzene rings with different conjugation degrees in order to synthesize CDs. The use of *o*-phenylenediamine was favorable to form conjugated sp^2^ domains and nitrogen-containing groups in the CD formation process.

In order to explore the influence of precursors on the fluorescence emission of the MCDs, three CDs were selected as typical samples in order to investigate the principle. According to the particular emission colors, they were named B-CDs, Y-CDs, and R-CDs, respectively. MCDs were obtained under the following conditions: *o*-phenylenediamine/[1,1′-binaphthalene]-2,2′-diamine = 1 at 180 °C for B-CDs, *o*-phenylenediamine/aniline = 1 at 180 °C for Y-CDs, and *o*-phenylenediamin/1,8-diaminonaphthalene = 1 at 180 °C for R-CDs, respectively.

**Figure 1:**
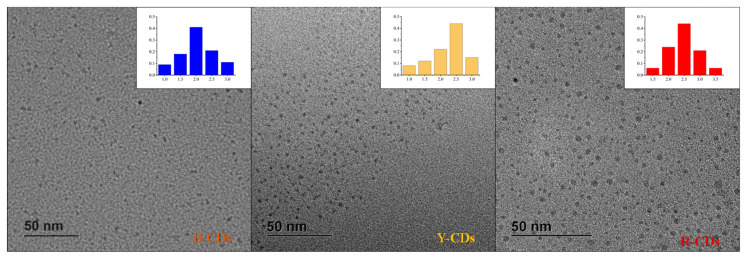
HRTEM images and the size distribution (insert) of MCDs.

The morphology and particle size of B-CDs, Y-CDs, and R-CDs were investigated by HRTEM, the results of which are shown in Figure 1. The three kinds of CDs were spherical and well dispersed, without obvious aggregation. The calculated average sizes of the B-CDs, Y-CDs, and R-CDs were 1.97 ± 0.24, 2.19 ± 0.32, and 2.51 ± 0.28 nm, respectively, and had a relatively uniform size distribution. 

Fourier-transform infrared (FTIR), Raman, X-ray powder diffraction (XRD), and X-ray photoemission spectroscopy spectra (XPS) analyses were also carried out to interpret the chemical compositions and surface functional groups of the prepared CDs. 

**Figure 2:**
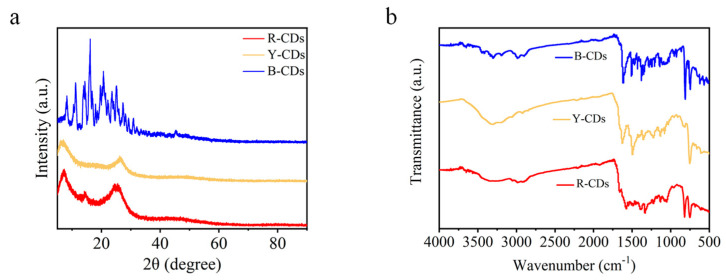
(**a**) XRD and (**b**) FTIR spectra of MCDs.

As shown in Figure 2a, compared with B-CDs, the XRD pattern of the Y-CDs and R-CDs expressed a minor sharp peak at 26.6°, which was due to the graphite structure with a layer spacing (002) of 0.41 and 0.34 nm [29]. The 26.6° peak was defined as the characteristic diffraction peak (002) of graphite. Its existence indicates the interlayer stacking between the CDs, which is like that of graphite (≈0.35 nm). This suggests that a similar accumulation (0.35 nm) of highly conjugated sp^2^ domains is dominant in CDs. At the edge of the CDs, the sp^3^ carbon or functional groups increase the inter-layer distance. The R-CD peak at 26.6° was the highest, thus predicting the greatest extent of graphitization; this suggested that R-CDs had a greater effective conjugation length than B-CDs and Y-CDs [30].

Figure S1 shows the Raman spectra of the prepared MCDs, which exhibited two peaks at 1354 cm^−1^ and 1581 cm^−1^, corresponding to the disordered (D band) and graphite (G band) of the carbon material. As we know, the ratio of I_D_/I_G_ equals the ratio of sp^3^/sp^2^ carbon, which represents the extent of graphite. The calculated ratio of I_D_/I_G_ was 0.91, 0.66, and 1.30 for B-, Y-, and R-CDs, respectively. A relatively lower I_D_/I_G_ for the B-CDs and Y-CDs indicated the much higher disorder degree of CDs.

In order to further explore the chemical structure of MCDs, the FTIR spectra of MCDs were tested, as shown in Figure 2b the board peak located at 3420 cm^−1^ was assigned to the N-H/O-H groups, the peak located at 2990 cm^−1^ was assigned to the -CH_2_ group, and the stretching vibrations for -C-C, -C=C, and C=O were located at 1340, 1500, and 1685 cm^−1^, respectively. These results indicated that the conjugated aromatic structure of the carbon core and the surface functional groups containing N and O (-COOH, -OH, -NH_2_) were incorporated successfully. At the same time, the gradually broadened C=O peak from the B-CDs, Y-CDs to R-CDs reflected the increase in carboxyl groups on the surface of MCDs.

To further determine the surface state and chemical composition of MCDs, XPS (X-ray photoelectron spectroscopy) was used to characterize the MCDs. As is evident in Figure S2, the full XPS spectra of MCDs showed three predominant peaks at 284.5 eV, 400 eV, and 532 eV, indicating the existence of C_1s_, N_1s_, and O_1s_, respectively. 

**Figure 3:**
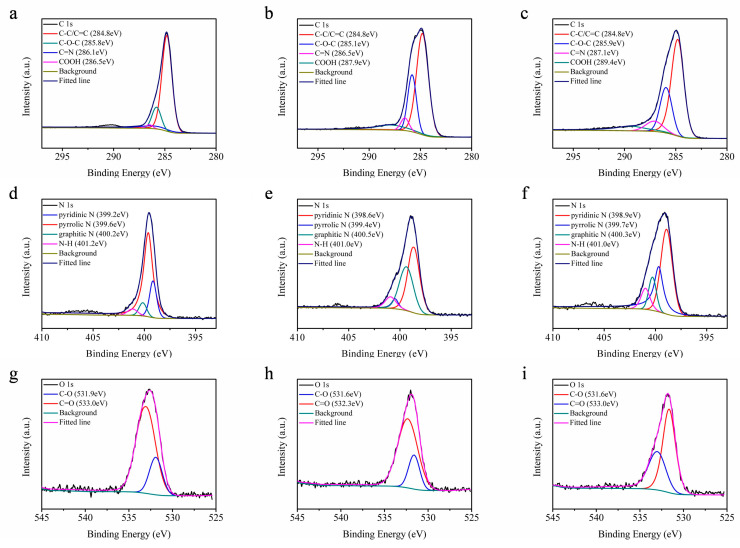
High-resolution XPS C_1s_ (**a**–**c**), N_1s_ (**d**–**f**) and O_1s_ (**g**–**i**) spectra of B-CDs, Y-CDs, and R-CDs.

Taking the high-resolution XPS spectrum of the R-CDs as an example, the high-resolution C_1s_ band (Figure 3a–c) exhibited four peaks at 284.8 eV for C-C/C=C, 285.01 eV for C-O-C, 285.83 eV for C=N, and 286.43 eV for -COOH. The high-resolution N_1s_ band (Figure 3d–f) of MCDs could be divided into pyridinic N (398.9 eV), pyrrolic N (399.68 eV), graphitic N (400.28 eV), and NH_2_ (400.97 eV). The high-resolution O_1s_ band (Figure 3g–h) was separated into two peaks at 531.46 eV and 532.86 eV, which were attributed to C-O and C=O. The binding energy and content of the specific groups of the three CDs are shown in Table S1.

With the increased number of benzene rings in the carbon precursors, the ratio of C to N changed from 2.41, 1.77 to 1.90, while the ratio of O/C increased from 0.29, 0.40 to 0.42 for B-CDs, Y-CDs, and R-CDs, respectively, which indicated that the increase in the oxygen amounts was caused by the presence of carboxylic groups [27]. The content of the carboxyl group significantly increased from B-CDs, Y-CDs, to R-CDs, which agreed with the FTIR result. When there was more surface oxidation present on the MCDs, there were more surface defects.

Meanwhile, the content of the graphitic N (C-N-C) group was increased from the B-CDs and Y-CDs to R-CDs, according to the XPS results. The existence of more C-N-C groups revealed that much more o-phenylenediamine was integrated with 1,8-diaminonaphthalene; this formed larger conjugate sp^2^ domains, as well as the effective conjugation length, thus resulting in the redshift in the emission. 

### 2.2. Spectral Properties of the Prepared CDs

**Figure 4:**
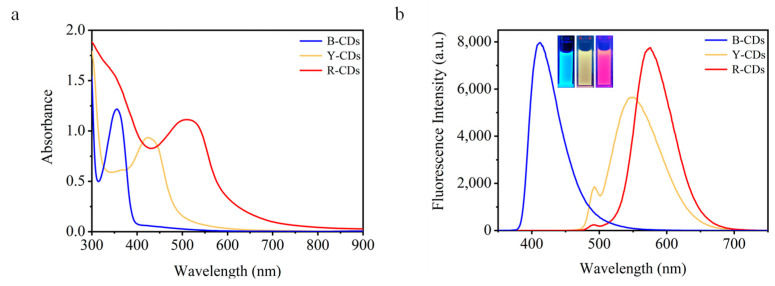
(**a**) The UV–vis absorption, (**b**) Fluorescence emission spectra of MCDs.

Figure 4a shows the UV–vis absorption spectrum of the prepared MCDs. B-CDs exhibited a strong absorption band at 360 nm, which was always ascribed to the n–π* transitions, conjugated to C=O and C=N. For Y-CDs, an absorption band was observed at 430 nm. The absorption peak of the R-CDs appeared at 500 nm. These peaks were considered as n-π* transitions in the aromatic sp^2^ system containing C=O and C=N bonds.

**Figure 5:**
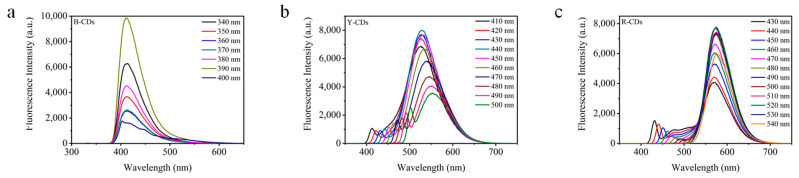
The fluorescence emission spectra of the (**a**) B-CDs, (**b**) Y-CDs and (**c**) R-CDs solutions at different excitation wavelengths.

As displayed in Figure 5, the fluorescence emission spectra of B-, Y-, and R-CDs at different excitation wavelengths were measured. The emission wavelength of the Y-CDs was dependent on the excitation wavelength. When the excitation wavelength of Y-CDs transformed from 410 to 500 nm, the emission wavelength gradually shifted from 520 to 555 nm. However, the emission peak of B-CDs was mainly 410 nm in the excitation wavelength range of 340~400 nm, indicating that it was an excitation-independent emission. In the excitation wavelength range of 430~540 nm, the emission range of R-CDs was longer, and the maximum emission peak was 580 nm. Different from Y-CDs, both B-CDs and R-CDs displayed excitation-independent emission behavior, which probably depended on their homogeneous surface states. Meanwhile, the excitation-dependent FL behavior was caused by the doping of graphitic nitrogen.

The optimal excitation and emission wavelength of B-, Y-, and R-CDs (Figure 4b) were 370/440 nm, 430/530 nm, and 520/580 nm, respectively. The optimal excitation wavelengths of these three CDs were basically consistent with their corresponding absorption bands, indicating that the emission mainly acted on the absorption bands from the n–π* transition of the C=O and C=N bonds of the aromatic sp^2^ system.

From the B-CDs and Y-CDs to R-CDs solution, the full width at half maximum (FWMH) was gradually decreased from 145 nm and 122 nm to 107 nm. Here, R-CDs possess a narrow FWMH, indicating the narrow band gap, which brought about the red emission.

We also optimized the reaction temperature (160, 180, 200 °C), reaction time (8, 10, 12, 14 h), and material ratio of o-phenylenediamin/1,8-diaminonaphthalene (1:2, 1:1, 2:1) at the pre-synthesis stage in order to obtain better fluorescence behavior. The fluorescence emission intensity of the R-CDs was investigated under these different conditions, and the results are shown in Figure S3. The optimal reaction conditions were a reaction temperature of180 °C, a reaction time of 12 h, and material ratio of o-phenylenediamin/1,8-diaminonaphthalene = 1:1.

For comparison, the original UV–vis and fluorescence emission spectra of *o*-phenylenediamine, aniline, 1,8-diaminonaphthalene, and [1,1′-binaphthalene]-2,2′-diamine. are presented in Figure S4. These four raw molecules had a very slight blue emission, and the absorptions in the UV–vis spectra were all in the UV range of 200–400 nm, which differed from the prepared MCDs; this verified that new luminescent centers were formed in the MCDs.

As the emission wavelength of B-, Y-, and R-CDs shifted from 440 nm, 530 nm to 580 nm, the size of the CDs slightly increased from 1.97 nm to 2.51 nm. Here, the redshift of the emission wavelength had a positive correlation with the size of the CDs [30]. This phenomenon suggested that maybe the increased effective conjugation length, the high extent of graphitization, and the extended sp^2^ domain gave rise to the increasing size. 

To further comprehend the photoluminescence, the fluorescence lifetime decay curves of the blue, green, and red CDs were measured at 375 nm using a multi-dimensional time-dependent single photon counting (TCSPC) method; the corresponding fitted equations with satisfactory goodness are shown in the supporting information. Using this method, the calculated average lifetimes (τ) were 5.08, 5.17 and 5.65 ns (Figure S5). In addition, it was found that the two radiative lifetimes τ1 and τ2 could be ascribed to the intrinsic recombination of the carbon core states and surface states [1]. Here, we can observe that the ratio of τ2 increased gradually with the redshift in the emission wavelength (Table S2), which indicated the existence of enhanced surface states. The relative quantum yield was measured by using Rhodamine B as a reference. The calculated *QY* of the B-CDs, Y-CDs, and R-CDs were 33%, 59%, and 11%, respectively. Here, the relative fluorescence quantum yield and fluorescence lifetimes were comparable with those reported for multicolor CDs, showing their excellent optical properties. In addition, we suggest that the non-radiative transition centers lead to the relatively low *QY* of R-CDs.

The photostability of the MCDs was also discussed. From Figure S6, we can see that the fluorescence intensity of the MCDs remained basically unchanged after continuous irradiation for 7200 s, showing their outstanding photostability and anti-photobleaching capabilities. Meanwhile, the appearance and fluorescence property of the CDs solution showed no obvious change after storage at room temperature for 3 months.

**Figure 6:**
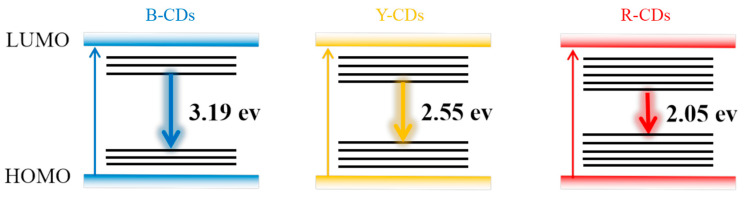
Dependence of the HOMO and LUMO energy levels of MCDs.

The energy gaps of the B-, Y-, and R-CDs were calculated using the formula E_gopt_ = 1240/λ_edge_, in which λ_edge_ is the maximum absorption edge wavelength. The HOMO–LUMO energy gaps of the B-, Y-, and R-CDs (Figure 6) were calculated as 3.19, 2.55, and 2.05 eV, respectively, corresponding to the redshift in the emission wavelength. This result was in accordance with the increase in the graphitic-N content.

Based on the above results, the redshift in the CDs emission was attributed to the increase of the graphite N content in the carbon core and the oxidation degree on the surface of the CDs. 

### 2.3. The Applications of MCDs

The prepared three CDs displayed hydrophilicity, the ability to be easily dissolved in various kinds of polarity organic solvents, such as DMSO, DMF, and dichloromethane, slight solubility in ethanol, but limited solubility in nonpolar organic solvents. 

**Figure 7:**
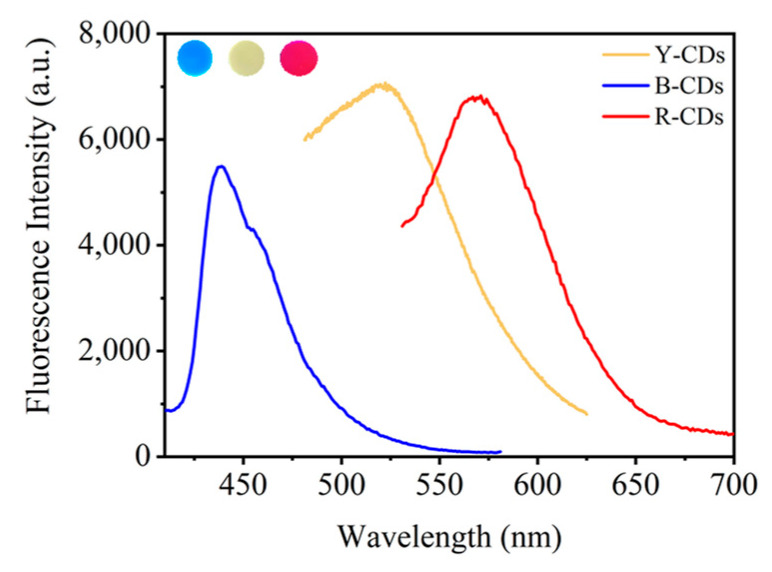
Fluorescence emission spectra of B-CDs/PVA, Y-CDs/PVA, R-CDs/PVA films. (Insert: The photographs of CDs/PVA films under UV lamp).

Several kinds of polymers can be used as solid-state luminescence matrices, such as poly-acrylic (PAA), poly-dimethylsiloxane (PDMS), and poly-vinylpyrrolidone (PVP). Considering that polyvinylpyrrolidone (PVA) has the advantages of a pure surface conformation, low cost, environmental friendliness, and good hydrophilicity, it was selected as the matrix to be used to fabricate CDs/PVA film. The B-CDs/PVA, Y-CDs/PVA, and R-CDs/PVA films exhibited bright blue, yellow, and red emissions under UV light irradiation, where aggregation and solid-state quenching can be effectively avoided. As shown in Figure S7, RCDs were well dispersed in the PVA matrix, and no obvious aggregation could be observed, thus proving that the PVA can be effective in preventing aggregation and fluorescence quenching of CDs. The fluorescence emission spectra of CDs/PVA films under the optimal excitation wavelength were depicted in Figure 7a. The optimal emission wavelengths were located at ≈440, ≈530, and ≈580 nm, respectively, which were the same wavelengths as the CDs in the solvents; this further confirms that the prepared CDs were homogeneously distributed in the PVA matrix with invariance property. The FWMHs of the B-CDs/PVA, Y-CDs/PVA, and R-CDs/PVA films were 111 nm, 104 nm, 85 nm, respectively, which were much lower than those of the CDs solution, indicating their potential to be applied in high color-purity displays and solid-state luminescence. 

**Figure 8:**
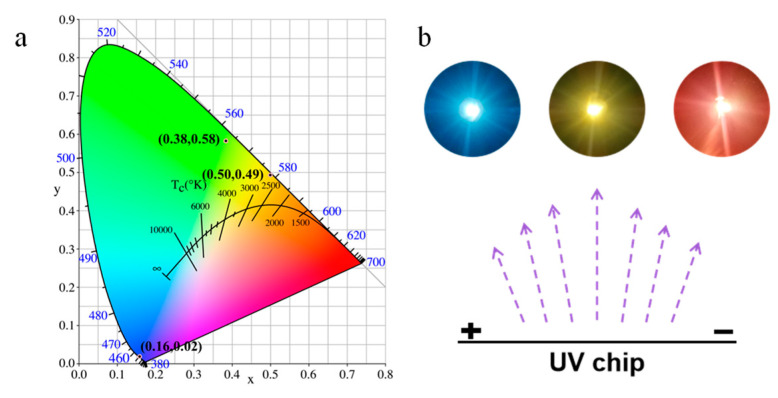
(**a**) The CIE color coordinates of the CDs/PVA based LEDs. (**b**) Optical photographs of the multicolor LEDs.

To further explore their practicability, the CDs/PVA films were used to package the LED chips. The CDs/PVA films were coated on UV chips to form illumination devices. These MCDs-based LEDs produced bright monochrome blue, yellow, and red light (Figure 8b), and the corresponding CIE coordinates were (0.16, 0.02), (0.38, 0.58), and (0.50, 0.49), based on the emission spectra of the device (Figure 8a). These CDs-based LEDs show great potential in the multicolor lighting field.

## 3. Experiments

### 3.1. Materials

N, N-dimethylformamide (DMF), dimethyl sulfoxide (DMSO), and polyvinyl alcohol (PVA) were ordered from Sinopharm Chemical Reagent Co., Ltd. (Shanghai, China). *O*-phenylenediamine, aniline, 1, 8-diaminonaphthalene, and 1, 1′-binaphthalene-2, 2′-diamine were ordered from Aladdin Chemical Reagent Co., Ltd. (Shanghai, China). All chemical materials had not been purified for analysis. The experimental water used was secondary deionized water (18 MΩ cm).

### 3.2. Characterizations

The size and morphology of the prepared MCDs were characterized by high-resolution transmission electron microscopy (TEM, JEOL JEM 2100F, Tokyo, Japan). The phase of the MCDs was characterized between 0° and 90° using 2θ scanning X-ray diffraction (XRD, Rigaku, Smart Lab 3KW, Japan). Fourier-transform infrared (FTIR) spectra were obtained on a spectrometer detector (Thermo, Nicolet is50, Madison, WI, USA). Raman spectra were measured on a confocal micro-Raman spectrometer detector (Thermo, DXR2, Madison, WI, USA). The MCDs prepared by the PHI1600EXCA photoelectron spectrometer were analyzed using X-ray photoelectron spectroscopy (Perkin-Elmer, USA). The ultraviolet–visible (UV–vis) absorption spectra of MCDs were implemented on a spectrophotometer (Purkinje, T9, Beijing, China). The fluorescence excitation/emission spectra of the MCDs were assembled using a fluorescence spectrophotometer (Hitachi, F-7000, Japan). The fluorescence decay curves were recorded on an FLS 1000 fluorescence spectrometer (Edinburgh Instruments, Livingston, UK).

### 3.3. Synthesis of CDs

**Scheme 1:**
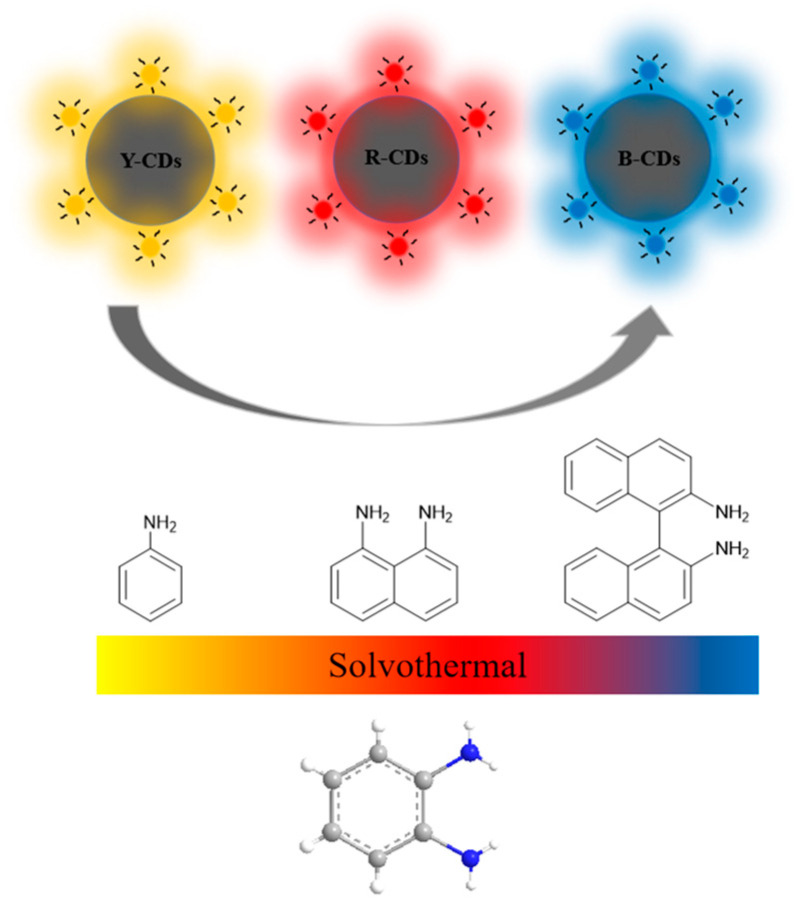
Schematic illustration for the preparation of MCDs.

As shown in Scheme 1, MCDs were composited using a simple solvothermal method. According to the procedure typically used to prepare R-CDs, o-phenylenediamine (0.25 mmol) and 1,8-diamino-naphthalene (0.25 mmol) were added to 10 mL of DMF. Then, the mixed solution was moved to a 100 mL Teflon-lined stainless-steel autoclave for a solvothermal pyrolysis reaction and heated at 180 °C for 12 h. After that, the autoclave was naturally chilled down to normal atmospheric temperature. Subsequently, the obtained MCD solution was dialyzed for 24 h through a 1000 Da dialysis membrane (molecular weight cutoff value) with ultrapure water to remove small molecules and unreacted chemical substances. Finally, the pure CD solution was kept at room temperature for further use. The purified CD solution was freeze-dried to obtain the CD dry powder, which was then ground and crushed into CD powder and characterized using XRD, FTIR and XPS.Y-CDs (precursor: 0.25 mmol *o*-phenylenediamine and 0.25 mmol aniline) and B-CDs (precursor: 0.25 mmol *o*-phenylenediamine and 0.25 mmol 1,1′-binaphthalene-2,2′-diamine) were obtained in the same way. 

### 3.4. The Determination of the Relative Fluorescence Quantum Yield and Fluorescence Lifetime

The relative fluorescence quantum yield of B-CDs, Y-CD, and R-CDs were measured by using Rhodamine B (*QYstd*: 89%) as a reference.

The *QY* can be calculated as follows:

(1) $$QY_{\chi }=QY_{std}\left(\frac{A_{std}I_{\chi }}{A_{\chi }I_{std}}\right){\left(\frac{\eta_{\chi }}{\eta_{std}}\right)}^{2}$$

where “*QY”* is the quantum yield, “*A*” is the absorbance at the maximum excitation wavelength, “*Ι*” represents the fluorescence integral area at the maximum excitation wavelength, “*η*” is the refractive index of the solvent, and the subscripts “*std*” and “*x*” indicate the reference substance and the substance to be measured, respectively. To reduce the impact of reabsorption, the absorbance of the measured substance at the maximum excitation wavelength was less than 0.05.

The fluorescence lifetime decay curves of B-CDs, Y-CDs, and R-CDs were measured at 375 nm excitation using a multi-dimensional time-dependent single photon counting (TCSPC) method, and the corresponding fitted equations and the calculated average lifetime (τ) equations are shown in the supporting information.

### 3.5. Preparation of CDs/PVA Films

In total, 4 g of PVA was dissolved in 16 mL of DMSO at 90 °C for 2 h in order to obtain a 20% PVA solution. Then, 10 mg of blue, yellow, and red luminous CD powder was added to each 20% PVA solution (20 mL) while the mixture was constantly stirred. The above solution was added to a self-made framework and naturally dried at room temperature for 24 h in order to obtain homogeneous CD/PVA films.

### 3.6. The Fabrication of CDs/PVA Composites-Based LEDs 

Commercially available LED chips (emission centers at 365 nm) with no phosphorus coating were purchased from Advanced Optoelectronics Technology Inc. Then, the blue luminous CD/PVA film was fastened to the top of the LED chip in order to manufacture the blue LED. Yellow LEDs and red LEDs were also prepared by using the above method. 

## 4. Conclusions

In summary, a new and simple synthetic procedure for preparing blue, yellow, and red emissive CDs was developed through the solvothermal method by using *o*-phenylenediamine with aniline, 1,8-diaminonaphthalene and [1,1′-binaphthalene]-2,2′-diamine as precursors, respectively. The emission properties of the CDs can be tuned by choosing carbon precursors with different amounts of benzene rings. Related characterizations demonstrated that the redshift of CDs was ascribed to the increasing effective conjugation degree, the high graphitization degree, and size. The prepared MCDs can be dissolved well in the polarity organic solvents, forming homogeneous solutions with bright blue, yellow and red emissions. A PVA polymer was used as the matrix for the fabrication of the CDs/PVA films. The films exhibited bright blue, yellow, and red emissions under UV light, as is also exhibited in organic solvents that effectively avoid aggregation and fluorescence quenching. By deposing these CDs/PVA films on UV chips, blue, yellow, and red LED devices with the CIE coordinates of (0.16, 0.02), (0.38, 0.58), and (0.50, 0.49) were successfully fabricated. Multicolor LEDs were constructed in order to demonstrate their potential applications in solid-state luminescence.

## Data Availability

All the data generated by this research are included in the article.

## Supplementary Materials

The following supporting information can be downloaded at: https://www.mdpi.com/article/10.3390/molecules28072957/s1, Figure S1: The Raman spectra of MCDs, (a) B-CDs, (b) Y-CDs, (c) R-CDs; Table S1. The content of specific groups in XPS results of MCDs; Figure S2. The XPS scan spectra of MCDs, (a) B-CDs, (b) Y-CDs, (c) R-CDs; Figure S3. The fluorescence emission spectra of R-CDs with different (a) reaction temperatures, (b) reaction times and (c) proportions of precursors excited by 520 nm; Figure S4. The UV–vis absorption spectra and fluorescence emission spectra of (a) *o*-phenylenediamine, (b) aniline, (c) 1,8-diaminonaphthalene, (d) [1,1′-binaphthalene]-2,2′-diamine. Figure S5. PL dynamics of MCDs; Table S2. The computational process of τ of the MCDs; Figure S6. The variation in fluorescence intensity for MCDs with irradiation time; Figure S7. The TEM image of R-CDs/PVA film.

## Sample Availability

Samples of the compounds are not available from the authors.
