# Supplementary material for: Blue, Yellow, and Red Carbon Dots from Aromatic Precursors for Light-Emitting Diodes
Source: Molecules. 2023 Mar 26;28(7):2957. doi: 10.3390/molecules28072957 (PMC10096300; doi:10.3390/molecules28072957)
Supplement: Supplementary file 1 [file molecules-28-02957-s001.zip › molecules-2291825-supplementary.pdf]

Supplementary Material

Blue, Yellow, and Red Carbon Dots from Aromatic Precursors for Light-Emitting Diodes

Liu Zhenzhen[1], Lu Xiaofei[1], Liu Menglin[2], Wang Wenjing[1]*

1 *College of Chemistry and Chemical Engineering; Shandong Sino-Japanese Center for Collaborative Research of Carbon Nanomaterials; Instrumental Analysis Center of Qingdao University; Qingdao University, Qingdao, 266071, P. R. China*

2 *Rizhao custom, Rizhao, Shandong, 276826, P. R. China*

---

* Corresponding author.

E-mail: wangwenjing@qdu.edu.cn.

Tel: 86-532-85953981.

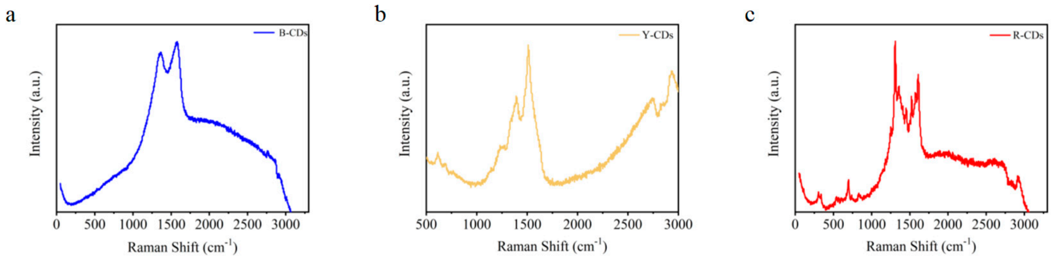

Figure S1. The Raman spectra of MCDs.

%	C-C/C=C	C-O-C	C=N	-COOH	pyridinic N	pyrrolic N	graphitic N	N-H
B-CDs	60.65	11.65	5.72	1.42	2.03	7.91	0.80	0.50
Y-CDs	43.27	20.74	2.80	9.24	8.73	6.89	1.22	1.49
R-CDs	45.61	4.22	9.75	17.78	7.711	5.48	1.94	1.52

Table S1. The content of specific groups in XPS results of MCDs.

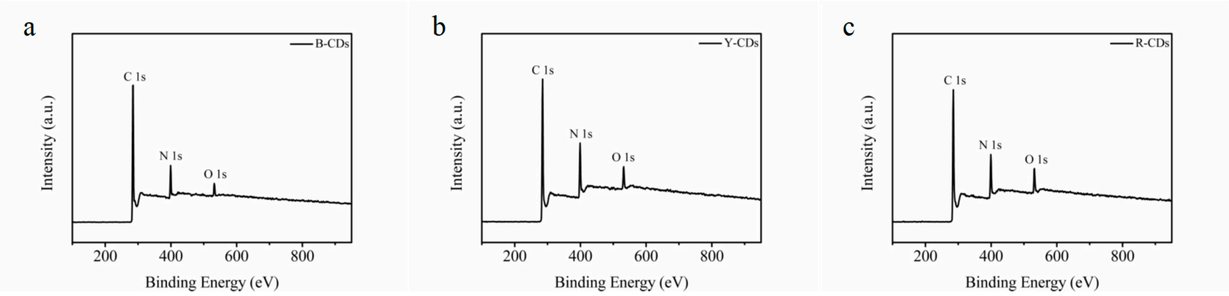

Figure S2. The XPS scan spectra of MCDs.

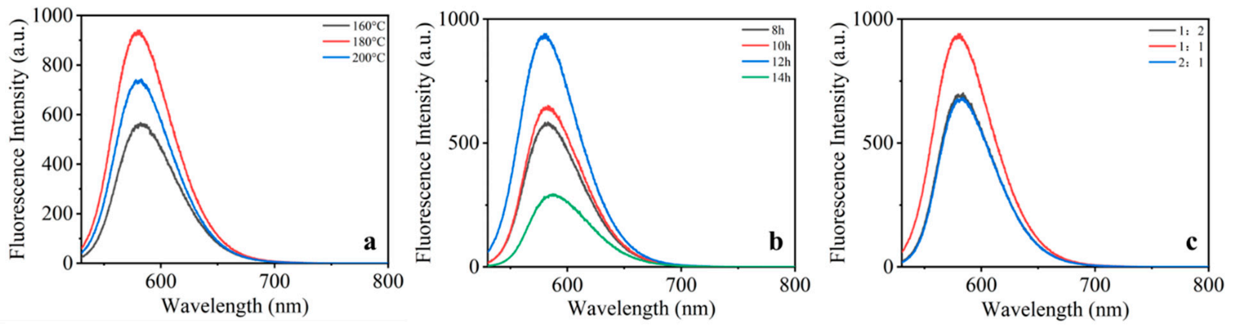

Figure S3. The fluorescence emission spectra of R-CDs with different (a) reaction temperatures, (b) reaction times and (c) proportions of precursors excited by 520 nm.

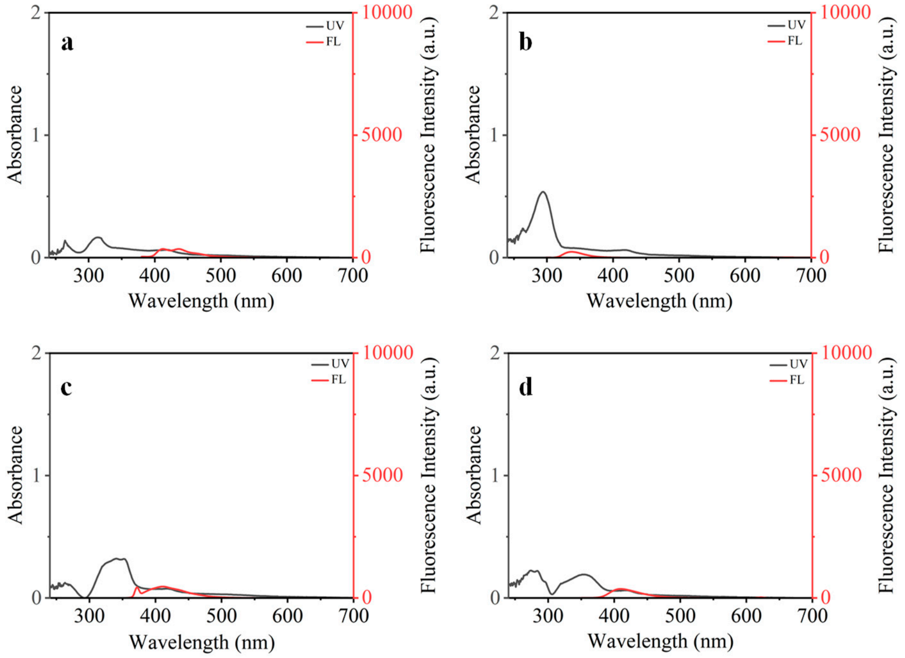

Figure S4. The UV-vis absorption spectra and fluorescence emission spectra of (a) *o*-phenylenediamine, (b) aniline, (c) 1,8-diaminonaphthalene, (d) [1,1'-binaphthalene]-2,2'-diamine.

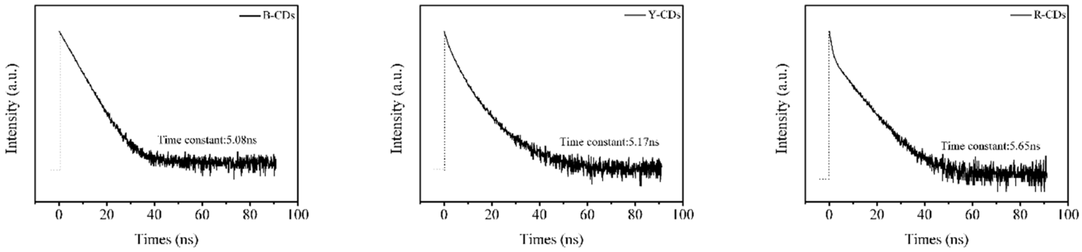

Figure S5. PL dynamics of MCDs.

Table S2. The computational process of $\overline{\tau}$ of the MCDs.

$\overline{\tau}$=B_1*τ_1+B_2*τ_2+B_3*τ_3+...							
B-CDs	B_1			τ_1			$\overline{\tau}$
	5.08 ns			100%			5.08 ns
Y-CDs	B_1	B_2	B_3	τ_1	τ_2	τ_3	$\overline{\tau}$
	1.36 ns	4.03 ns	9.72 ns	13.25%	60.57%	26.18%	5.17 ns
R-CDs	B_1	B_2		τ_1	τ_2		$\overline{\tau}$
	0.94 ns	7.20 ns		24.81%	75.19%		5.65 ns

According to the below equation and related parameters in Table S2.

$$R(t) = B_1e^{(-t/\tau_1)} + B_2e^{(-t/\tau_2)} + B_3e^{(-t/\tau_3)}$$

The fitted equation and χ^2 of B-CDs, Y-CDs, and R-CDs are as follows:

B-CDs: $R(t) = 3067.4211e^{(-t/5.0797)}$, $\chi^2 = 1.1867$

Y-CDs: $R(t) = 1105.7166e^{(-t/1.3629)} + 1707.7139e^{(-t/4.0340)} + 306.4238e^{(-t/9.7184)}$,

$$\chi^2 = 1.2222$$

R-CDs: $R(t) = 2346.7903e^{(-t/0.9449)} + 932.7779e^{(-t/7.2042)}$, $\chi^2 = 1.2186$

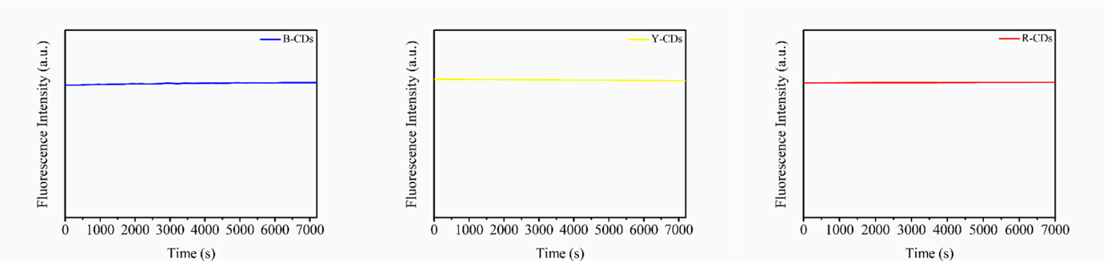

Figure S6. The variation of fluorescence intensity for MCDs with irradiation time.

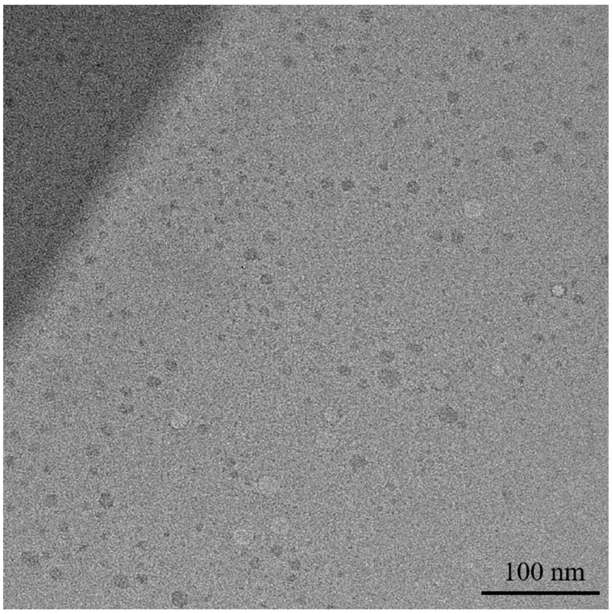

Figure S7. The TEM image of R-CDs/PVA film.
